# Safety and efficacy of immune checkpoint inhibitor cancer therapy in patients with preexisting type 1 diabetes mellitus

**DOI:** 10.3389/fendo.2023.1242830

**Published:** 2023-10-31

**Authors:** Robin Hilder, Karen Tsai, Zoe Quandt, Dayna Isaacs, Alexandra Drakaki, Yan Xing, Gino K. In, Trevor E. Angell, Melissa G. Lechner

**Affiliations:** ^1^ Department of Medicine, University of California, Los Angeles (UCLA)-Olive View Health System, Los Angeles, CA, United States; ^2^ Department of Medicine, University of California, Los Angeles (UCLA) Geffen School of Medicine, Los Angeles, CA, United States; ^3^ Division of Endocrinology, City of Hope Comprehensive Cancer Center, Duarte, CA, United States; ^4^ Division of Diabetes, Endocrinology and Metabolism, Department of Medicine, University of California, San Francisco, San Francisco, CA, United States; ^5^ Division of Hematology and Oncology, Department of Medicine, University of California, Los Angeles (UCLA) Geffen School of Medicine, Los Angeles, CA, United States; ^6^ Division of Oncology, City of Hope Comprehensive Cancer Center, Duarte, CA, United States; ^7^ Division of Hematology and Oncology, Department of Medicine, Keck School of Medicine of University of Southern California (USC), Los Angeles, CA, United States; ^8^ Division of Endocrinology and Diabetes, Department of Medicine, Keck School of Medicine of University of Southern California (USC), Los Angeles, CA, United States; ^9^ Division of Endocrinology, Department of Medicine, University of California, Los Angeles (UCLA) Geffen School of Medicine, Los Angeles, CA, United States

**Keywords:** immune checkpoint inhibitor, type 1 diabetes mellitus, autoimmune endocrinopathy, immune related adverse events, safety

## Abstract

**Introduction:**

Immune checkpoint inhibitors (ICI) produce dramatic tumor shrinkage and durable responses in many advanced malignancies, but their use is limited by the development of immune-related adverse events (IRAEs) that occur in up to 60% of patients and often affect endocrine organs. Concern for more severe IRAEs in patients with preexisting autoimmune diseases, including type 1 diabetes mellitus (T1DM), has led to the exclusion of such individuals from clinical trials of ICI therapy. As a result, little is known about the safety and efficacy of ICI in this population. Here, we report safety and treatments outcomes in ICI-treated patients with preexisting T1DM.

**Methods:**

This retrospective case-controlled study evaluated adult patients with T1DM who received ICI therapy for solid malignancies from 2015 to 2021 at four academic medical centers. Patients with prior ICI therapy, bone marrow transplantation, or pregnancy were excluded. We collected data on demographics, cancer diagnosis and treatment, IRAE incidence and severity, and diabetes management. Controls were matched 2:1 by age, sex, cancer diagnosis, and ICI therapy class.

**Results:**

Of 12,142 cancer patients treated with ICI therapy, we identified 11 with a preexisting confirmed diagnosis of T1DM prior to starting ICI therapy. Mean age was 50.6 years, 63.6% were women, and most received anti-PD1/PDL1 monotherapy (10/11) compared with combination therapy (1/11). Grade 3/4 IRAEs were seen in 3/11 subjects with preexisting T1DM and were hepatitis, myositis, and myasthenia gravis. All three cases had interruption of ICI therapy and administration of adjunct therapies, including steroids, IVIG, or mycophenolate mofetil with resolution of the IRAE. The odds of all-grade IRAEs and of severe IRAEs were comparable between cases and controls matched for age, sex, cancer type, and ICI therapy [OR 0.83 (95% CI 0.2–3.56), p = 0.81, and OR 1.69 (0.31–9.36), p = 0.55, respectively]. Overall survival was not different between patients with T1DM and controls (p = 0.54). No patients had hospitalizations for diabetes-related complications during therapy.

**Discussion:**

These data suggest that ICI monotherapy can successfully be used in patients with preexisting T1DM, with IRAE rates comparable with individuals without preexisting T1DM. Larger, prospective studies of these potentially life-saving ICI therapies that include patients with preexisting autoimmunity are warranted.

## Introduction

Immune checkpoint inhibitors (ICI), including anti-programmed death protein (PD1) or ligand (PDL1) and anti-cytotoxic T lymphocyte antigen (CTLA4) agents, have revolutionized cancer therapy, producing dramatic tumor shrinkage and durable responses in many types of advanced malignancies (1). Indeed, nearly half of US cancer patients are now eligible for ICI cancer therapy (2). Unfortunately, the increased immune activation from ICI therapies that is desired for tumor cell killing can also lead to the development of unwanted autoimmunity in healthy tissues. Such immune-related adverse events (IRAEs) can limit the use of ICI cancer therapy and contribute to patient hospitalizations, organ damage, and even premature death. With the expanding use of ICI therapies in cancer patients, the diagnosis and management of IRAEs have emerged as important clinical problems.

Due to a perceived concern that patients with preexisting autoimmunity may be at increased risk for developing IRAEs or that ICI may exacerbate an existing autoimmune disease, these patients were excluded from most cancer immunotherapy clinical trials (3). Even after Food and Drug Administration (FDA) approval of ICI agents, many physicians and patients have continued concerns about the safety of these treatments in individuals with preexisting autoimmune disease. Indeed, several recent studies have demonstrated that even patients with subclinical autoimmunity, such as those with thyroid autoantibodies but no thyroid dysfunction, had a significantly higher risk of developing hypothyroidism during ICI treatment compared to patients with no thyroid autoimmunity (4). While most IRAEs are reversible, including those classified as grade 3/4, ICI-induced endocrinopathies are usually permanent, including T1DM. ICI-induced thyroid dysfunction may be readily managed or produce only mild symptoms. In these cases, the exclusion of patients with advanced malignancies from ICI therapy may in fact lead to poorer clinical outcomes due to cancer progression. Thus, the decision to use ICI therapies in patients with preexisting autoimmunity is nuanced and must balance the anticipated risks and benefits of cancer treatment.

Endocrine tissues are among the most frequently affected by both spontaneous and ICI-associated autoimmunity (5). While the cause of IRAEs remains poorly understood, it is likely driven by a combination of cytotoxic T-cell and B-cell activation with the production of pro-inflammatory cytokines (6, 7). Several studies have reported on the incidence of IRAEs in patients treated with ICI therapies for advanced malignancies with pre-existing autoimmune conditions. These conditions include rheumatologic, gastrointestinal, neurologic, and dermatologic autoimmune diseases. Evaluation of patients with preexisting endocrine autoimmunity has focused on thyroid autoimmunity, with few cases of T1DM in the literature. Depending on the autoimmune condition and its definition in each study, the rates of IRAEs and outcomes have been highly variable, resulting in sometimes conflicting recommendations on the use of ICI therapy in these patients. One retrospective study of 52 patients with melanoma who had preexisting autoimmunity reported that IRAEs were mild, were manageable, and did not require discontinuation of ICI therapy (8). In contrast, a study evaluating ICI in patients with preexisting inflammatory bowel disease (IBD) reported a fourfold increased risk of severe gastrointestinal adverse events compared to patients without underlying IBD (9). Similar studies report that preexisting autoimmunity is associated with a significantly increased risk of grade 2 IRAEs but not grade 3 or 4 IRAEs when compared to control populations (10, 11). A systematic review and meta-analysis of 11 observational studies reporting 868 patients with cancer and various preexisting autoimmune conditions found that 40% of patients had no autoimmune flare or *de novo* IRAEs, concluding that ICIs can be safely used in these patients (12). Similarly, 619 ICI-treated patients with preexisting autoimmunity in 14 publications reported IRAEs of any grade at 60% (95% CI = 52%–68%) but were often manageable without the need to discontinue therapy (13). It is reported that 11.73% of IRAEs in patients with preexisting autoimmunity resulted in the cessation of ICI and 3.5% required hospitalization and immunosuppressive treatments (14, 15). The variable rates and relative lack of data focused on individuals with T1DM therefore provide the impetus for our study. Here, we report the outcomes for safety and efficacy of ICI therapy in individuals with preexisting T1DM across four academic medical centers, compared with controls matched for age, sex, cancer diagnosis, and ICI treatment. These data will help guide clinicians in counseling their patients on the safety and tolerability of ICI therapies with preexisting autoimmune diabetes.

## Methods

### Study design

This retrospective, case-controlled multicenter study evaluated adult patients aged 18 years and older who received one or more FDA-approved ICI therapies for malignancies between 2015 and 2021 at four academic medical centers in the United States. Sites included the University of California, Los Angeles Health System (UCLA), Los Angeles, CA; the University of Southern California Keck Medical Center and Los Angeles County General Medical Center (USC), Los Angeles, CA; the City of Hope Comprehensive Cancer Center (COH), Duarte, CA; and the University of California, San Francisco Health System, (UCSF), San Francisco, CA. Institutional review board approval was obtained at each site (COH 23043-241543; UCLA 20-000857; USC: HS-19-00304; UCSF: 17-22987).

### Patients

This study evaluated patients with histologically confirmed diagnoses of solid malignancies who underwent treatment with ICI therapy. Patients were included if they received ICIs as first-line monotherapy, combination therapy, or second-line therapy after receiving non-ICI cancer treatments, including chemotherapy, radiotherapy, and vaccine therapy. Eligible patients included those treated with at least one of the following FDA-approved regimens: nivolumab, pembrolizumab, atezolizumab, durvalumab, avelumab, or ipilimumab. Exclusions were pregnancy, administration of prior ICI therapies, and bone marrow transplantation. Cases were patients with a diagnosis of T1DM prior to starting ICI therapy. Patients with preexisting T2DM or a new diagnosis of T1DM after receiving ICI therapy were excluded.

International Classification of Disease (ICD)-10 codes were used to search the electronic medical records (EMR) at each medical center to identify patients with a diagnosis of T1DM who had received an ICI medication. ICD-10 codes E10.9 (type 1 diabetes mellitus without complications) and E10.65 (type 1 diabetes mellitus with hyperglycemia) were the most common categories for identifying T1DM cases. Specifically, cases were patients who had a formal diagnosis of T1DM prior to starting an ICI. Charts were screened by manual chart review for inclusion. Data on the presence of autoantibodies (anti-GAD65, IA2, insulin antibodies) were not available prior to the start of ICI therapy in this retrospective study. Diagnoses of T1DM after ICI therapy were excluded as this could not be distinguished from ICI-associated T1DM. IRAEs were classified according to the criteria outlined in the NIH CTCAE Version 5 and cumulatively reported as crude incidence. IRAEs were categorized according to the organ or system reported.

Controls were selected in a 2:1 ratio to cases from the cohort of patients without preexisting autoimmunity at each site and matched for sex, age (within 10 years), cancer, and ICI class (anti-PD1/PDL1 monotherapy or combination anti-CTLA4 + anti-PD1/PDL1 therapy). In two cases, a matched control was not available within the same institutional cohort and therefore an appropriate control was selected from another site.

### Data extraction

Data extraction was independently performed in January 2023 by one investigator at each site. A standardized data collection template was used to obtain patient demographic characteristics including sex, age at cancer diagnosis, age at ICI start, and age deceased if applicable. Additional data were obtained on comorbidities and history of autoimmunity, Eastern Cooperative Oncology Group (ECOG) Performance Status Scale at diagnosis and follow-up, cancer type, stage, location of metastases at diagnosis, tumor PDL1 expression, history of prior chemotherapy, ICI class and dates administered, and response [complete response (CR), partial response (PR), stable disease (SD), progressive disease (PD)]. We obtained data regarding the types of IRAE reported, how long after initiating ICI they developed, grade, and outcome with dose and duration of adjunct immunomodulatory therapies. High-dose steroids were classified as doses greater than prednisone 20 mg, or equivalent conversions, for >2 weeks. We gathered data on T1DM insulin regimen, changes during therapy, the use of digital wearables for T1DM, and if the patient had any presentations or hospitalizations for hyperglycemia.

### Outcomes

The primary outcome for this study was the safety of ICI in patients with and without preexisting T1DM. We defined this as the incidence of clinically significant, treatment related, adverse effects (IRAE grade 3 or higher) according to the NIH CRCAE Version 5.0, and the outcomes of IRAEs. This included whether IRAEs resulted in ICI interruption or discontinuation and need for adjunct immunomodulatory therapies. We evaluated ICI efficacy by clinical response as documented in the medical chart according to the Response Evaluation Criteria in Solid Tumors (RECIST) scale (16). Overall survival was calculated from date of cancer diagnosis to date of last follow-up at the time of data extraction, or documented date of death. The secondary outcome was defined as worsening glycemic control in patients with preexisting T1DM, specifically requiring hospitalization or presentation for medical evaluation of hyperglycemia or the complications of hyperglycemia.

### Statistical analyses

All patients were included in the assessment of baseline characteristics by descriptive analysis to summarize characteristics at diagnosis and the start of ICI treatment. We considered subgroups, including cancer type, ICI class (anti-PD1/PDL1 monotherapy versus combination anti-PD1/PDL1 and anti-CTLA-4 therapy) for data extraction to the overall cohort. Unpaired two-sided Welch’s t-test without assumption of equal variances was used to compare differences in normally distributed continuous variables, whereas Mann–Whitney U test was used for non-parametric variables. Chi-square or Fisher’s exact test was used to compare differences between proportions and categorical variables, with odds ratio (OR) determined using the Baptista–Pike method. Conditions for performing chi-square calculations were not met in all subgroup analyses; therefore, qualitative analysis was performed for evaluating incidence of IRAE by grade and organ system. Survival was compared between cases and controls by log-rank test. For all, significance was defined as alpha = 0.05, with correction for multiple comparisons. Statistical analyses were performed using Prism software (v9.4, GraphPad).

## Results

### Patients

Of 12,142 patients with solid malignancies who were treated with ICI therapies across four academic health systems, we identified 11 cases with preexisting type 1 diabetes mellitus. The mean age of cancer diagnosis was 50 years (SD 13.83), and 7/11 were women (63.6%) (**Table 1**). Case diagnoses included cervical adenocarcinoma (1), breast (3), head and neck squamous cell carcinoma (1), renal cell carcinoma (1), ovarian (1), and lung (4). Among the patients with T1DM who received ICI therapies, at the time of initiating ICI therapy, all 11/11 (100%) had stage III/IV disease compared to controls 16/22 (72.73%) had stage III/IV disease (p 0.077 = 95% CI 0.00–0.99). Five of 11 cases (45.5%) had more than two metastatic lesions at diagnosis. In addition to T1DM, 6/11 cases (54.5%) had further coexisting autoimmune conditions, including hypothyroidism (5/11), asthma (1/11), and Celiac disease (1/11). There were 10 of 11 cases that received treatment with single-agent anti-PD1 or anti-PDL1 therapy, whereas only one received combination anti-PD1 + anti-CTLA4 treatment. ICIs were used as first-line therapy in five cases (45.5%), and second-line treatment after chemotherapy in six cases (54.6%) due to treatment failure or as a maintenance therapy. The median duration of ICI therapy was 3 months (IQR 2.5–6).

**Table 1:** Demographic and cancer diagnosis data for cases and controls.

	Case, n (%)	Control, n (%)	p-value
Patients	11	22	
Age at cancer diagnosis (years)			
Mean (std. deviation)	50.64 (13.83)	52.09 (13.62)	0.77
Age at ICI initiation (years)			
Mean (std. deviation)	52.91 (14.45)	54.45 (13.51)	0.77
Sex			
Male	4 (36.4%)	8 (36.4%)	1.00
Female	7 (63.6%)	14 (63.6%)	
Primary tumor			
Cervical 1.00	Vadenocarcinoma	1 (9.1%)	2 (9.1%)
Lung	4 (36.4%)	8 (36.4%)	
Breast	3 (27.3%)	6 (27.3%)	
Head and neck squamous cell	1 (9.1%)	2 (9.1%)	
RCC	1 (9.1%)	2 (9.1%)	
Ovarian	1 (9.1%)	2 (9.1%)	
Stage at ICI initiation			
I/II	0	6 (27.27%)	0.077
III/IV	11 (100%)	16 (72.73%)	

ICI, immune checkpoint inhibitor. CI, confidence interval.

Control subjects (n = 22) were matched for age, sex, cancer type, and ICI therapy (**Tables 1**, **2**) Notably, two of 22 controls (9.1%) had preexisting autoimmunity, documented as hypothyroidism and ulcerative colitis. The median duration of ICI therapy in controls was 5 months (IQR 3–11) and was not significantly different from cases (p = 0.40).

**Table 2:** Cancer treatment type and duration for cases and controls.

	Case, n (%)	Control, n (%)	p-value
Received prior chemotherapy	6 (54.6%)	13 (59.1%)	
ICI first-line monotherapy	3 (27.3%)	3 (13.6%)	
ICI first-line combination therapy	2 (18.3%)	6 (27.3%)	
ICI drug			
Nivolumab	2 (18.2%)	2 (9.1%)	0.9152
Durvalumab	1 (9.1%)	1 (4.5%)	
Pembrolizumab	6 (54.5%)	15 (68.2%)	
Atezolizumab	1 (9.1%)	2 (9.1%)	
Ipilimumab + nivolumab	1 (9.1%)	2 (9.1%)	
Duration of ICI therapy			
<3 months	4 (36.4%)	7 (31.8%)	0.3515
3–6 months	5 (45.5%)	6 (27.3%)	
6–12 months	0 (0.0%)	5 (22.7%)	
>12 months	2 (18.2%)	4 (18.2%)	

ICI, immune checkpoint inhibitor.

### Incidence of moderate and severe IRAEs

Data for the incidence of all grade IRAEs were collected, and qualitative analyses were used to evaluate the type according to the CTCAE Version 5.0 (**Table 3**). Two of 11 cases had documented grade 1 or 2 IRAEs (18.2%), comprising one patient with ICI thyroiditis requiring thyroid hormone replacement and one patient with preexisting hypothyroidism requiring an increased dose of thyroid hormone replacement. Both patients continued ICI therapy without interruption. Three of 11 cases experienced a grade 3 or 4 IRAE (27.3%) with details of each case provided in **Suppl. Table 2**. These cases included one patient with hepatitis (transaminase elevation) from pembrolizumab, one with hepatitis and myositis from durvalumab, and one with myasthenia gravis with myositis from nivolumab. All three patients with grade 3 or 4 IRAE required permanent discontinuation of ICI therapy and initiation of adjunct immune modulating therapies which included prolonged courses of high-dose steroids in all three patients, intravenous immunoglobulin (IVIG) in two patients and mycophenolate mofetil in one patient.

**Table 3:** Incidence of IRAEs during ICI therapy for cases and controls.

	Case, n (%)	Control, n (%)	OR (95% CI)
All grade IRAEs	5 (45.5%)	11 (50.0%)	0.83 (0.2–3.56)
Grades 1 and 2	2 (18.2%)	7 (31.8%)	0.48 (0.08–2.81)
Grade 3 and 4	3 (27.3%)	4 (18.2%)	1.69 (0.31–9.36)
Organ system			
Gastroenterological	2 (18.2%)	0 (0.0%)	
Musculoskeletal	2 (18.2%)	0 (0.0%)	
Neurologic	1 (9.1%)	0 (0.0%)	
Respiratory	0 (0.0%)	1 (4.5%)	
Cardiac	0 (0.0%)	1 (4.5%)	
Endocrine	0 (0.0%)	1 (4.5%)	
Dermatologic	0 (0.0%)	1 (4.5%)	

IRAEs, immune-related adverse events; OR, odds ratio; CI, confidence interval.

Odds of IRAEs were not statistically significantly different between cases and controls for all grades.

In comparison, seven of 22 controls had a documented grade 1 or 2 IRAE (31.8%), including thyroiditis (two subjects), hypophysitis with secondary adrenal insufficiency (2), arthritis (1), hepatitis (1), and tenosynovitis (1). Four of 22 controls had a reported grade 3 or 4 IRAE (18.2%), which included one subject each with pneumonitis, pericarditis, thyroiditis, and cutaneous eruption. All IRAEs resulted from anti-PD1 therapy, except for hypophysitis occurring during combined ipilimumab and nivolumab therapy. Among grade 3 or 4 IRAEs, two patients required permanent discontinuation of ICI therapy and one patient required temporary interruption of ICI therapy. No cases or controls developed grade 5 IRAEs.

IRAEs developed at a median of 16.5 weeks (range 6–30 weeks) of starting ICI therapy in cases, compared with 18 weeks (range 3–121 weeks) in controls. More than half of all IRAEs occurred within the first 6 months of ICI therapy in both cases and controls (3/5 cases vs. 6/11 controls). The odds of all-grade IRAEs and of severe IRAEs (grade 3 or higher) were comparable between cases and controls matched for age, sex, cancer type, and ICI therapy [OR 0.83 (95% CI 0.2–3.56), p = 0.81, and OR 1.69 (0.31–9.36), p = 0.55, respectively] (**Table 3**).

### Tumor response to ICI therapy

**Figure 1:**
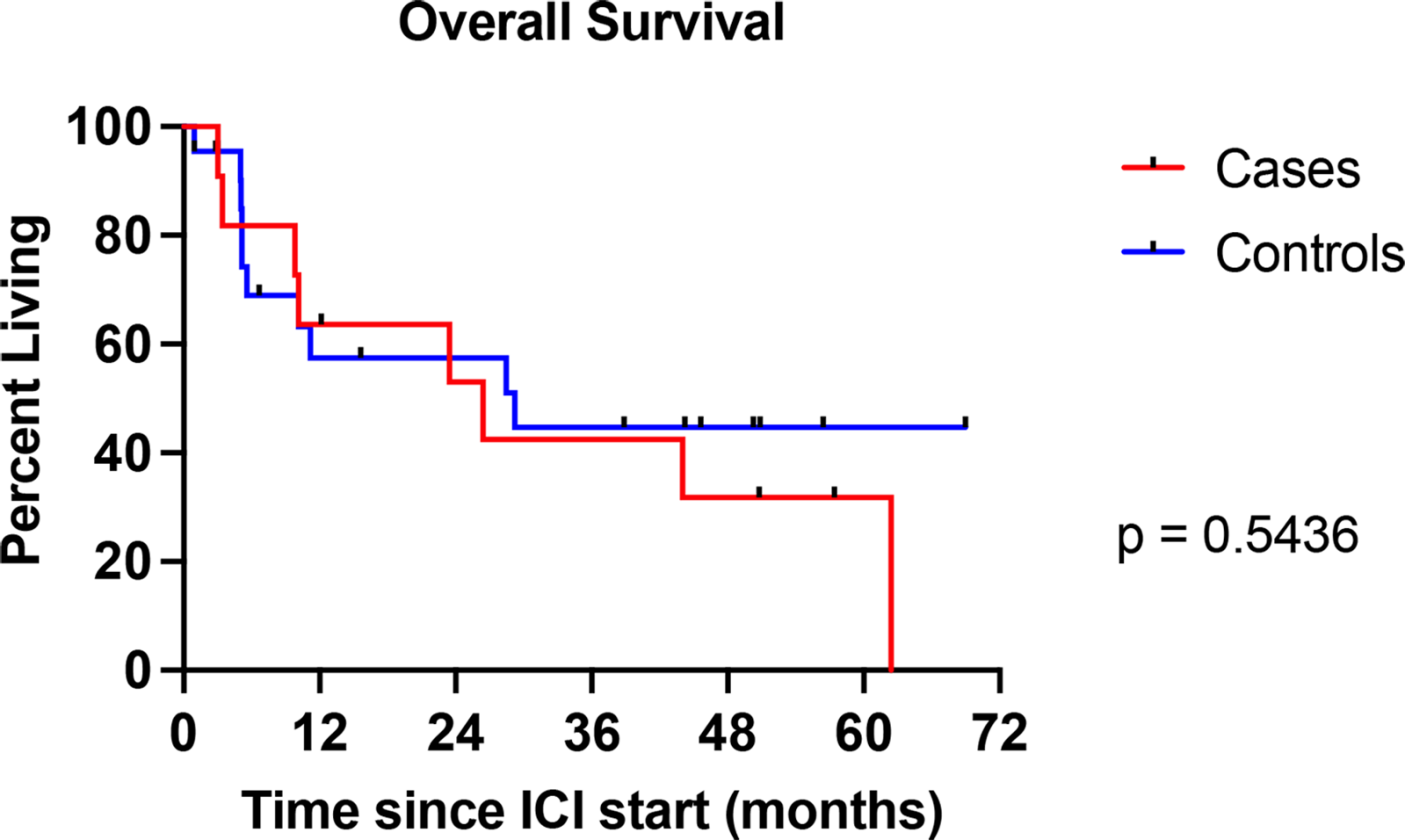
Kaplan–Meier curve showing overall survival for individuals with preexisting type 1 diabetes mellitus (T1DM) (cases) compared with individuals without T1DM (controls). Curve comparison by log-rank test.

We evaluated patient response to ICI therapy using RECIST criteria. Among cases, nearly half had a measurable tumor response, including three (27.3%) with CR and two (18.2%) with PR. The remaining patients had progressive disease (6/11, 54.5%) (**Table 4**). All patients with grade 3/4 IRAEs had a partial or complete tumor response. By comparison, tumor responses in controls were 18.2% (4/22) CR, 40.9% (9/22) PR, 9.1% (2/22) SD, and 31.8% (7/22) PD. Overall survival was not significantly different at timepoints <1, 1–3, and >3 years (chi-square, p = 0.7661) (**Table 4**), or over time (**Figure 1**, log-rank p = 0.436) between cases and controls. Taken together, these data suggest that tumor responses to ICI therapy in patients with T1DM are similar to individuals without preexisting T1DM.

**Table 4:** Tumor response and survival for cases and controls during ICI therapy.

	Case, n (%)	Control, n (%)	p-value
Tumor response on ICI by RECIST criteria			0.3362
Complete response	3 (27.3%)	4 (18.2%)	
Partial response	2 (18.2%)	9 (40.9%)	
Stable disease	0 (0.0%)	2 (9.1%)	
Progressive disease	6 (54.5%)	7 (31.8%)	
Tumor response in subjects with grade 3+ IRAEs			0.4594
Complete response	2 (66.7%)	1 (25.0%)	
Partial response	1 (33.3%)	2 (50.0%)	
Stable disease	0 (0.0%)	1 (25.0%)	
Progressive disease	0 (0.0%)	0 (0.0%)	
Overall survival			0.7661
>3 years	4 (36.4%)	7 (31.8%)	
1–3 years	2 (18.2%)	3 (13.6%)	
<1 year	5 (45.5%)	10 (45.5%)	
Unknown	0 (0.0%)	2 (9.1%)	

IRAEs, immune-related adverse events; RECIST, Response evaluation criteria in solid tumors.

### Glycemic control and use of diabetes technology

Aside from incident autoimmune side effects, another clinical concern in the use of ICI therapy in patients with T1DM is the impact on glycemic control. While not routinely used for the treatment of endocrine IRAEs, systemic glucocorticoids remain a mainstay for the management of IRAEs in other tissues, including ICI-associated colitis and pneumonitis. Importantly, glucocorticoids promote the development of hyperglycemia, namely, by increasing lipolysis, proteolysis, and gluconeogenesis, and reducing insulin-mediated glucose uptake and glycogen synthesis in skeletal muscle (17). These mechanisms when exacerbated in patients with T1DM can profoundly increase the risk of developing hyperglycemia and even precipitate diabetic ketoacidosis (DKA). Thus, it was important to investigate the impact of ICI therapy and its associated supportive care on glycemic control in our cases with T1DM. Eight of 11 T1DM cases (72.7%) utilized an insulin pump while on ICI, including 5/11 (45.5%) with a concurrent continuous glucose monitor (CGM) (**Table 5**). No patients used a CGM alone, and three patients were managed with multiple daily insulin injections. Regarding steroids, 5/11 T1DM patients (45.5%) received scheduled single-dose steroids as part of their immunotherapy regimens and 3/11 (27.3%) received high-dose glucocorticoid therapy (equivalent to prednisone 20 mg/day or higher for more than 2 weeks) for the treatment of IRAEs. Overall, 6/11 patients had a documented increase in their daily insulin requirements (54.5%) while receiving ICI therapy. One patient restarted insulin after rejection and failure of a pancreatic transplant for T1DM following development of IRAE-mediated grade 4 myasthenia gravis with myositis. Notably, there were no ED visits, hospitalizations, or presentations to medical services for management of hyperglycemia or its complications. In summary, patients with preexisting T1DM could be managed with insulin adjustment and use of usual care, including diabetes technology and insulin therapy, during ICI treatment without adverse outcomes related to glycemic control.

**Table 5:** Glycemic management, complications, and use of diabetes technology in subjects with T1DM during ICI therapy.

	Case, n (%)	Control, n (%)
Use of diabetes technology		
CGM	5 (45.5%)	0
Insulin pump	8 (72.7%)	0
None	3 (27.3%)	0
Change in insulin regimen during ICI		
Yes	6 (54.5%)	0
No	3 (27.3%)	0
Not documented	2 (18.2%)	0
ED visit or hospitalization for T1DM		
Yes	0 (0%)	0
No	11 (100.0%)	0
ED visit or hospitalization		
Yes	0 (0%)	0
No	11 (100.0%)	0

CGM, continuous glucose monitor; ED, emergency department.

## Discussion

With the increasing use of immune checkpoint inhibitors for cancer therapy, the specter of unwanted autoimmune side effects in healthy tissues remains a concern for many physicians and patients, particularly those with preexisting autoimmune disease. As we navigate how to use these therapies safely and most effectively in this patient population, more data are needed to guide our clinical recommendations. Here, we report the incidence and outcomes of IRAEs in a carefully defined cohort of patients with preexisting T1DM who received ICI therapies and provide comparison with a matched group of patients without T1DM. A strength of our study was well-defined criteria for T1DM to identify cases by manual chart review in combination with automated data extraction from electronic medical records, rather than reliance upon ICD codes alone. This approach proved more accurate than ICD coding to identify patients with preexisting T1DM (**Suppl. Table 1**). We report a similar incidence of all grade and severe (grade 3 or higher) IRAEs in patients with preexisting T1DM compared with matched controls without T1DM. Because of the small number of cases identified in our study, reflecting the rarity of T1DM patients who have been treated with ICI therapy, it is also valuable to compare the rate of IRAEs in our group to the rate of IRAEs reported in larger studies across a general population. The rate of overall and severe IRAEs observed in our cases and controls was similar to previously published cohorts of ICI-treated patients. One of the largest studies in melanoma, for example, reported an incidence of severe IRAEs of 59% with combination nivolumab plus ipilimumab therapy and 28% in the nivolumab alone group (18).

These data provide an important complement to prior medical coding-based studies in patients with preexisting autoimmunity. In patients without known autoimmune conditions, there is a wide range in the published rates of IRAEs by trial, ICI class (combination versus monotherapy), and malignancy. A recent meta-analysis evaluating the incidence of IRAEs in 206 patients with preexisting autoimmunity, including rheumatologic, dermatologic, endocrine, and gastrointestinal disorders, found that 62.1% of patients with preexisting autoimmunity experienced an IRAE of any grade compared with 51.9% of patients without preexisting autoimmunity (19). While data specific to T1DM were not provided, the findings are consistent with our results showing a similar incidence of IRAEs in patients with T1DM compared to controls. Another meta-analysis evaluating 619 patients with preexisting autoimmune conditions who received ICI found a pooled incidence of autoimmune disease flares and *de novo* IRAEs of 60% (95% CI 52%–68%) (13). One notable feature that differentiates T1DM from other autoimmune conditions is that diagnosis coincides with complete loss of gland function (insulin dependence), such that autoimmune disease flares do not exacerbate clinical disease or impact the underlying management. Therefore, in contrast to perhaps gut or joint autoimmunity, concern for exacerbation of autoimmune activity against pancreatic beta cells should not impede the use of ICI in patients with T1DM.

Furthermore, our data suggest that insulin therapy can be safely continued in these patients while on ICI treatment, including through steroid treatment for other IRAEs, without adverse outcomes related to hyperglycemia or DKA. We observed that patients using both insulin pump and subcutaneous insulin injection regimens were able to continue their pre-ICI regimens with controlled titrations in daily insulin requirements. No patients were hospitalized for DKA or complications of hyperglycemia while receiving ICI therapy. Technology like CGM and insulin pumps facilitate tighter glycemic control and may have contributed to the absence of adverse glycemic outcomes in this modern-era study.

In deciding whether to offer a cancer therapy, physicians weigh the expected risks with expected benefits. All cases with T1DM who received ICI were diagnosed with advanced malignancies (i.e., stage III or IV disease) (**Table 1**), whereas nearly one-third of controls were offered ICI therapy for early Stage malignancies (i.e., stage I or II). This may reflect clinical hesitancy to employ checkpoint inhibitors for cancer therapy in individuals with autoimmunity risk who have less advanced cancers. In addition, our study found similarly favorable clinical responses to ICI treatment in these individuals. Patients with preexisting T1DM had similar PR and CR rates, as well as overall survival, to matched controls. These data, while from a small cohort, suggest that ICIs are not only as safe but also as effective for tumor control, in patients with T1DM as the more general population of individuals treated with ICIs. Interestingly, endocrine autoimmunity during ICI treatment has been associated with improved antitumor responses. This association is best established for ICI thyroiditis, a common IRAE. A systematic review and meta-analysis of 47 studies on thyroid dysfunction and ICI efficacy concluded that in multiple malignancies, the development of a thyroid specific IRAE was associated with improved overall survival (OS) and progression-free survival (PFS) (OS: HR 0.52, CI 0.43–0.62, p < 0.001; PFS: HR 0.58, CI 0.50–0.67, p < 0.001). Whether this beneficial relationship of tumor response extends to other preexisting autoimmune endocrinopathies remains unclear and warrants further study.

Our study is inherently limited by its retrospective nature and by a small number of cases, despite engagement of multiple large academic cites and screening of over 12,000 ICI-treated patients. However, our data provide important information about ICI safety in patients with T1DM and supports the inclusion of these patients in future immunotherapy trials while we await the conclusion of ongoing prospective studies. In addition, documentation of IRAEs was retrospective and done as part of usual care and therefore reporting of IRAEs may have been underreported. We attempted to mitigate this bias by comparing matched controls from the same academic centers for our outcomes.

## Conclusion

Taken together, our data suggest that ICI therapies can be used in cancer patients with preexisting T1DM, with rates of IRAEs comparable to patients without T1DM. These patients, like all patients treated with ICI therapies, should be counseled on the risk of IRAEs. Physicians should be aware of the need to titrate insulin regimens through the course of therapy. Our findings suggest CGM and insulin pump technologies can be safely used in individuals with T1DM on ICI therapy and may help to ease the burden of glycemic monitoring and insulin administration. Specific challenges for glycemic control that can be anticipated during ICI therapy include hyperglycemia with systemic steroid therapy given for the treatment of IRAEs in other tissues and a change in appetite and food intake with gut IRAEs. Importantly, our study found that patients with T1DM had clinical tumor responses to ICI therapy that were similar to those in controls without preexisting T1DM matched for age, sex, cancer type, and immunotherapy regimen. Larger, prospective studies inclusive of patients with preexisting T1DM are needed to further define the pattern of IRAEs during ICI treatment in patients with preexisting autoimmunity to ensure the equitable and evidence-based use of these potentially life-saving cancer therapies.

## Data availability statement

The original contributions presented in the study are included in the article/**Supplementary Material**. Further inquiries can be directed to the corresponding authors.

## Ethics statement

The studies involving humans were approved by institutional review boards at the University of California, Los Angeles Health System (UCLA, 20-000857), Los Angeles, CA; the University of Southern California Keck Medical Center and Los Angeles County General Medical Center (USC, HS-19-00304), Los Angeles, CA; the City of Hope Comprehensive Cancer Center (COH, 23043-241543), Duarte, CA; and the University of California, San Francisco Health System, (UCSF, 17-22987), San Francisco, CA. The studies were conducted in accordance with the local legislation and institutional requirements. The ethics committee/institutional review board waived the requirement of written informed consent for participation from the participants or the participants’ legal guardians/next of kin because this was a retrospective chart review study.

## Author contributions

ML, TA, KT, ZQ, AD, DI, and RH contributed to the conception and design of the study. RH, DI, KT, TA, GI, ZQ, and YX performed data collection. RH coordinated the data collection. RH, ML, and TA performed the statistical analysis. RH wrote the first draft of the manuscript. All authors contributed to the manuscript revision, read, and approved the submitted version.
