# Supplementary material for: Safety and efficacy of immune checkpoint inhibitor cancer therapy in patients with preexisting type 1 diabetes mellitus
Source: Front Endocrinol (Lausanne). 2023 Oct 31;14:1242830. doi: 10.3389/fendo.2023.1242830 (PMC10643762; doi:10.3389/fendo.2023.1242830)
Supplement: Supplementary file 1 [file Table_1.pdf]

	Subjects with cancer who received ICI treatment	Subjects with any ICD code for T1DM	Defined cases with pre-existing T1DM after manual chart review
UCLA	3,130	45	3
USC	1,578	3	1
COH	4,545	13	2
UCSF	2,889	76	5
**Total**	**12,124**	**137**	**11**

Supplemental Table 1. Summary of T1DM case identification from each participating site. UCLA, University of California, Los Angeles Health System; USC, University of Southern California Keck Medical Center and Los Angeles County General Medical Center; COH, City of Hope Comprehensive Cancer Center; UCSF, University of California, San Francisco Health System; ICI, immune checkpoint inhibitor therapy; T1DM, type 1 diabetes mellitus; ICD, International Classification of Diseases.

Case	Sex	Age at Cancer Diagnosis	Pre-Existing Autoimmune Disease	Cancer	Metastases	ICI Line of Therapy	ICI Regimen	Tumor Response	IRAE Type	IRAE Grade	IRAE Treatment	Immunotherapy Cessation
1	M	78	T1DM	Squamous Cell Lung	Liver, Biliary	2	Durvalumab	CR	Hepatitis, Myositis	3	Steroids, IVIG	Yes
2	F	37	T1DM	Lung Adeno-carcinoma	Lung, Brain	1	Pembrolizumab	PR	Hepatitis	3	Steroids, Mycophenolate	Yes
3	F	47	T1DM, Hashimoto's thyroiditis	Renal Cell Carcinoma	Brain, Kidney, Liver, Lungs	1	Nivolumab	CR	Myasthenia Gravis, Myositis	3	Steroids, IVIG	Yes

Supplemental Table 2. Summary of Grade 3 & 4 IRAEs and their outcomes reported in case subjects. IRAEs, immune related adverse events, ICI, immune checkpoint inhibitor. T1DM, type 1 diabetes mellitus; ICI, immune checkpoint inhibitor; CR, complete responses; PR, partial response; IVIG, intravenous immunoglobulin
